# Unilateral orchidectomy in donkey (Equus asinus): Evaluation of different surgical techniques, histological and morphological changes on remaining testis

**Published:** 2013

**Authors:** Magda Mahmoud Ali Omar, Khaled Mohamed Ahmed Hassanein, Abdel-Razek Khalifa Abdel-Razek, Haroon Ali Yousef Hussein

**Affiliations:** 1 *Department of Animal Surgery, Faculty of Veterinary Medicine, Assiut University, Assiut, Egypt; *; 2 *Department of Pathology and Clinical Pathology Faculty of Veterinary Medicine, Assiut University, Assiut, Egypt; *; 3 *Department of Theriogenology, Faculty of Veterinary Medicine, Assiut University, Assiut, Egypt.*

**Keywords:** Animal, Histology, Leydig cells, Testis, Unilateral orchidectomy

## Abstract

Unilateral orchidectomy (UO) is required when further breeding potential is important. It is sometimes necessary to remove a single testis in a matured stallion for therapeutic reasons. In this study, twelve donkeys were used to evaluate three techniques of unilateral castration, histological and morphological changes on the remaining testis. Results of the study showed that each of the surgical techniques used had its advantages and disadvantages in comparison with the other two techniques. Therefore the selection among the three techniques depends on the surgeon preferences and the environment in which the unilateral orchidectomy is performed. The volume of the remaining testis recorded at the end of the study was significantly greater than that estimated at the start of the study (*p < *0.05). The percentage of sperm motility obtained from the remaining testis was significantly decreased (*p < *0.05). Histological examination of the testis in open surgery (group I) (where the scrotum was left opened) revealed severe hemorrhages, edema and fibrosis. The test is showed degenerative changes in the seminiferous tubules and interstitial orchitis. Histological examination of the testes removed using a closed technique, (in groups II and III) where the scrotum wound was sutured, revealed hyperplasia of spermatogenic series and Leydig cells. In conclusion, unilateral orchidectomy had compensatory effects on the weight and volume of remaining testis. Adverse effects on sperm motility and viability can affect the fertility of the animal.

## Introduction

Castration is the most common surgical procedure performed by equine veterinarians.^1^ The reason most often cited for castration in animals is to reduce masculine behavior in animals unsuitable for the genetic pool and, less commonly, due to testis neoplasia, trauma, inguinal hernias or torsion of the spermatic cord.^2^^,^^3^

Unilateral orchidectomy (UO) is required when further breeding potential is important. It is sometimes necessary to remove a single testis in a matured stallion for therapeutic reasons, such as testicular tumors, varicocele, hydrocele, testicular trauma, orchitis, periorchitis, spermatic cord torsion and most cases of scrotal hernia.^4^^,^^5^ The diseased testis is removed, whilst the healthy one is retained to preserve the fertility of the stallion and this is referred to as UO.^3^^,^^6^ Removing a testis for any reason results in compensatory hypertrophy and increased sperm production in the remaining testis.^7^^,^^8^ The testis and epididymal weight are shown to increase linearly with age and by UO and, tubular diameter and epithelial height were greater in UO than in stallions.^4^

When performing orchidectomy, the scrotal or inguinal incision can be sutured or it can be left open to heal by second intention. The decision is based on many factors. 

The purpose of this study was to evaluate three different surgical techniques of UO in donkeys, as well as the histological and morphological changes on the remaining testis. 

## Materials and Methods

**Animals. **Twelve donkeys, 4-10 years old weighing 100 to 230 kg, were included in this study. The animals were kept in a clean, well ventilated, warm, lightened stable, and had free access to food and water. Careful palpation of the scrotum and testes of each donkey confirmed the presence of two normally descended testes. The scrotum and the testes were normal in size and the testes were freely movable inside the scrotum. Ethical approval used for all procedures was obtained from a local ethics committee acting under guidance from Faculty of Veterinary Medicine, Assiut University, Assiut, Egypt.

**Anesthesia and operation protocols. **Aseptically**, **castration was carried out under the effect of intramuscular xylazine HCl (Adwia pharmaceuticals Co., Cairo, Egypt) 0.5 mg kg^-1^ and local infiltration anesthesia using 2% lidocaine HCl (El-Nasr Pharmaceutical Chemicals, Kaliobeya, Egypt) injected at the site of incision inside the scrotum subcutaneously. The animals were divided into three groups each of four animals. The donkeys were restrained in lateral recumbency and the upward hind limb was held in cranially fixed position. In group I, the scrotal incision was left open after castration to heal by second intension. In group II, the scrotal incision was sutured to allow primary wound closure of the scrotal skin, and in group III, unilateral scrotal ablation was performed and the remaining scrotal incision was sutured. 

**First operation**

**Technique. **On the day of surgery the donkeys were kept away from food and water from the previous night. They were restrained in lateral recumbency. The upward hind limb was held in cranially fixed position to allow sufficient working area in the inguinal region. Scrotal circumference and length of each testis were recorded using a caliper. The scrotum was prepared for aseptic surgery using chlorhexidine scrub solution. With 10 mL of 2% lidocaine hydrochloride a ring block was given at the proximal third of the scrotal neck, followed by injection of 5 mL as a linear infiltration at the site of incision. 

In group I: An incision parallel to the median raphe was made on the cranial aspect of the scrotum of the left testis. The incision was passed through the whole layers of the scrotum and the parietal tunic of the testis based on technique described by Cox.^7^ The testis was prolapsed through this incision and pulled away from the body. A fenestration was made through the mesorchium and the vascular portion of the cord. For crushing of the blood vessels, an equine emasculator was placed on the spermatic cord for 5 min on a distance approximately 5 cm proximal to the testis; the vascular portion of the cord was ligated with placement of catgut No. 1 (Yangzhou Honesty International Co. Ltd., Jiangsu, China). Ligature into the crushed zone of the spermatic cord. After ligation, the testis was removed with a scissor. The vaginal tunic was then closed with the same suture material. The scrotal incision was left open to facilitate drainage from the wound site.

**Fig. 1:**
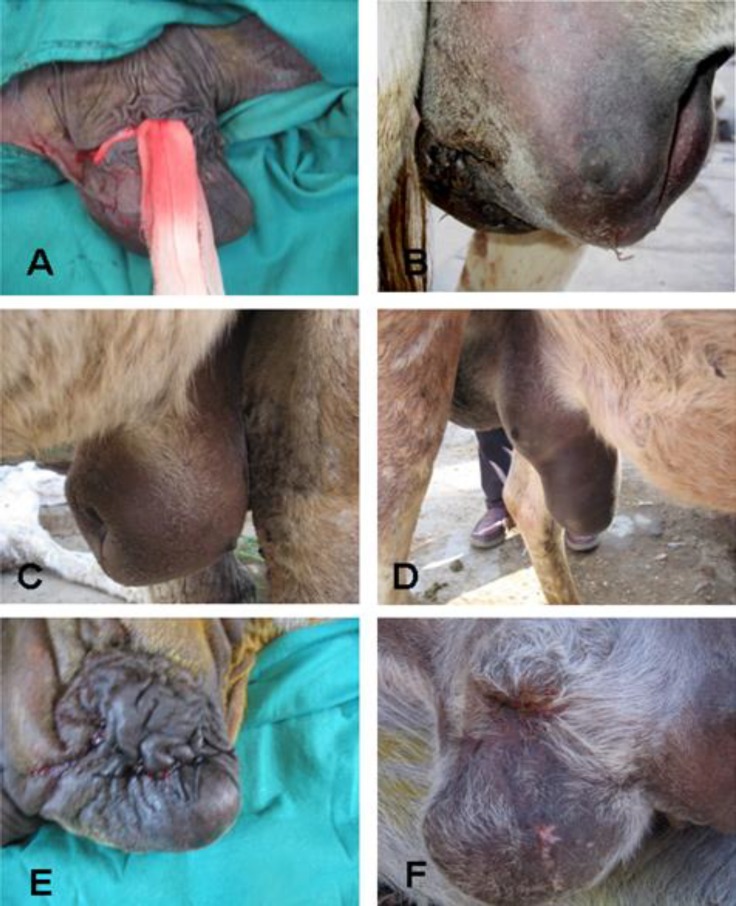
**(A** **)** The last stitch of the scrotal incision was not knotted and a drain was applied through it to allow drainage of the exudates. **(B, C, D)** Swelling of the scrotal area after suturing the scrotal incision in groups II and III. **(E, F)** The final appearance of the scrotal wound of group III was better in its final appearance in comparison with group II two weeks after surgery

Group II**:** The testis was removed in a same manner to group I, but after removal of the testis, the dead space of the scrotal sac in this side was obliterated with a simple continuous suture pattern using No. 3 vicryl (Yangzhou Honesty International Co. Ltd., Jiangsu, China). The subcutaneous tissue was also closed in the same technique. The skin was sutured with simple interrupted sutures using No. 0 vicryl. These were not removed but were left to resorb. A piece of gauze was applied through the distal end of the scrotal wound to allow drainage of the exudate; this drain was changed daily for the first three days following operation (Fig. 1A).

Group III: A circular incision was made near the base of the scrotal portion around the left testis passing through all layers of the scrotum. After ligation of the spermatic cord, the testis was removed with removal of all the surrounding tissues and skin around it. The wound was sutured and a drain was placed in as described in group II.

**Post-operative care. **Following castration, the donkeys were received 3000 IU of anti-tetanic serum (Ishita Pharma, Maharashtra, India) intramuscularly and procaine benzylpenicillin (Pfizer Animal Health, Giza, Egypt) 18 mg kg^-1^ intramuscularly. 

The scrotal incision of donkeys in group I was rinsed to remove excessive crusted exudates and then digitally reopened and explored. The drained scrotal cavity was then rinsed massively. The changes in donkey behavior and the size of the swelling at the site of surgery were monitored daily. In group II and III the drain was changed daily for the first week post castration. 

**Measurements. **The removed testis was used as a control for the corresponding one for certain parameters; the removed testis was undergone the following examinations: Testicular weight (TW) with and without the epididymis, epididymal semen parameters measure for the percent of sperm motility, and the percent of live sperms. Liberated sperms from minced epididymal tail in physiological saline solution were examined. Motility percentage was tested using phase contrast microscope. Percentage of abnormal sperm and percentage of live sperm were estimated from a film stained by eosin and nigrosin. 

**Second operation.** After 120 days, the donkeys of all groups were subjected to orchidectomy of the remained testis. The dimensions of the remained testis were measured by caliper; weight of the removed testis and epididymis was estimated, and semen liberated from the epididymal tail was treated as the first one. 

**Post-operative care. **Management procedures for the next few days included hosing the outside of the scrotum to remove obvious contamination. Chlorhexidine ointment 1% (Clipper Distributing Company, LLC, St. Joseph, Mo, USA) was applied topically to the inside of both hind legs to prevent skin scalding from the scrotal discharge.

**Measurements** **. **The volume of the testis was calculated from ellipsoid equation. The difference between the volume of right and left testes was compared. The volume of removed testis in the second stage of study was compared with its volume at the start of the experiment. The difference in weights between the two testes was statistically studied. The characteristic of sperm from each testis were investigated. 

**Histology. **Sections from each testis were fixed in 10% neutral buffered formalin, dehydrated in a graded alcohol series, cleared with methyl benzoate and embedded in paraffin wax. Sections of 5 μm were cut and stained with hematoxylin and eosin (H & E).^8^ Stained sections were examined under light microscope (Model CX31, Olympus, Tokyo, Japan) and photographed using digital camera (Model Camedia C-5060, Olympus, Tokyo, Japan).

**Computerized morphometric analysis. **Spermatogonia, spermatocytes, spermatid, spermatozoa and Sertoli cells were counted in 10 cross section of the test is using transmitted light, bright-field microscope (Model Axiostar plus, Carl Zeiss, Oberkochen, Germany). 

**Statistical analysis: **Statistical analysis was carried out by analysis of variance (ANOVA) and the results were compared using Student *t*-test. The level of significant difference was 5%, probability value of less than 0.05 was considered statistically significant. A *p*-value of less than 0.01 was considered highly significant (*p <* 0.01), *p*-value of less than 0.001 was considered extremely significant (*p* < 0.001). All statistical tests were performed using Graphpad Software Package (Graphpad software Inc. San Diego, CA, USA).

## Results

**Anesthesia. **The local anesthesia provided by lidocaine in combination with xylazine was enough in performing the castration without any anesthetic risk or complications and the animals were able to stand directly after the operation. No anesthetic complications were recorded during or after surgery. 

**Operative and post-operative complications**

**Group I.** Slight swelling of the scrotum was apparent the day after each orchidectomy but began to subside after three days. Then the swelling was subsided gradually. Scrotal discharge continued for one week after castration and the wound began to decrease gradually in size at the end of the first week of castration. Daily flushing of the scrotal wound was very important to remove debris and flies accumulated inside the wound cavity. Hemorrhage occurred in two animals in this group within the 48 hr post operation, and was completely controlled with gauze packing the scrotal cavity for 24 hr. Two animals in group I and one animal in group III showed cautious gait during the first week of operation. Healing period required for the wound in this group was about three weeks.

**Groups** **II and III.** Severe (group II) and mild (group III) swellings of the scrotum and the surrounding area were observed in all animals of these two groups. The swelling was extended forward on the ventral aspect of the abdomen and reached the umbilicus. It resulted in pressure on the prepuce and urethra causing difficulties in urination for the first three days following orchidectomy in three animals in group II (Figs. 1B, 1C, and 1D). This swelling subsided spontaneously within one week after castration. Hand walking of the animals and massage of the swollen area with daily change of the drain were important in decreasing the size of the swelling. 

Ablation of the scrotum in group III resulted in smaller swelling at the area of the scrotum and better wound healing appearance in comparison with group II (Figs. 1E and 1F).

**Measurements. **The mean volume of the left and right testes ± SE at the beginning of study were 87.96 ± 6.70 mL and 89.87 ± 5.95 mL, respectively. The right testis volume recorded at the end of study (127.28 ± 16.7 mL) was significantly more than that of estimated at the start of study (79.86 ± 5.95 mL) for the left one (*p* < 0.05). Volume of left testis and right one at time of removal were significantly different (*p* < 0.05). The weight of the removed testes was 105.36 ± 7.70 g and 134.50 ± 13.82 g for the right and left ones, respectively. The dissected left and right epididymis weights were 20.20 ± 1.87 g and 23.93 ± 2.53 g, respectively. The motility percentages of sperm obtained from the remained right epididymis tail (84.00 ± 2.91) were significantly lower (*p* < 0.05) than that obtained from the left one (73.00 ± 3.39). The total abnormalities percentages were 23.26 ± 1.34 for sperm obtained from left epididymal tail and 29.36 ± 1.22 for the right one and the difference was significant (*p* < 0.01).

**Fig. 2:**
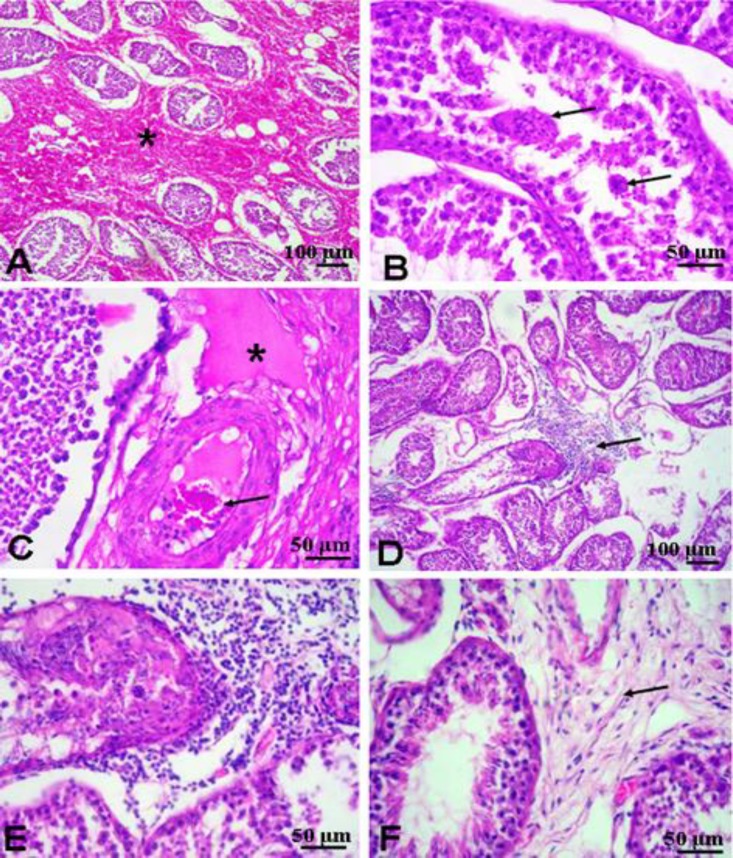
Representative micrographs for histological findings in the testis of open surgery group. **(A)** Hemorrhage (asterisk). **(B)** Testicular degeneration with formation of spermatid giant cells (arrows). **(C)** Edema of the interstitial tissue (asterisk). **(D & E)** Interstitial orchitis with focal area of lymphocytic infiltration (arrow). **(F)** Interstitial fibrosis (arrow) (H & E).

**Fig. 3:**
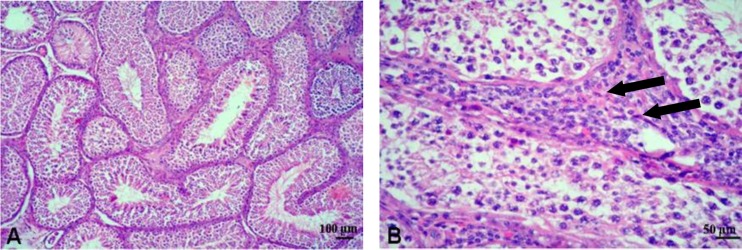
Representative micrographs for histological findings in the testis of closed surgery group **(A & B)** Hypertrophy of the testis, notice hyperplasia of Leydig cells (arrows) (H & E).

**Histology. **Histological examination of the testis in group I revealed severe hemorrhages (Fig. 2A). The seminiferous tubules showed degenerative changes and characteristic multinucleated giant cells in the lumen of seminiferous tubules (Fig. 2B). Interstitial edema was also seen (Fig. 2C). Moreover, degeneration of the testis and interstitial orchitis were observed. The inflammatory reaction was in the form of heavy infiltration of lymphocytes (Figs. 2D and 2E). Moreover, fibrosis of the testis was also noticed in the interstitial tissue (Fig. 2F). Examination of H & E-stained sections of the testis in group II revealed hypertrophy of the seminiferous tubules and hyperplasia of the Leydig cells (Figs. 3A and 3B).

**Computerized morphometric analysis. **The mean concentration of Sertoli cells, spermatogonia, spermato-cytes and spermatozoa in open surgery was extremely significantly decreased (*p < *0.001) when compared with the control group. The mean concentration of Sertoli cells and spermatogonia in the closed surgery groups (Group II and III) was highly significantly increased (*p* < 0.01) when compared with control group. In addition, there was no significance (*p* > 0.05) of the mean concentration of spermatid and spermatozoa when compared with the control group (Table 1).

**Table 1:** The mean value of spermatogenic series in studied groups (Mean ± SD).

**Spermatogenic series**	**Group I**	**Group II and III**	**Control**
**Sertoli cells**	43.70 ± 2.62***	95.00 ± 5.970**	73.14 ± 2.86
**Spermatogonia**	310.50 ± 24.10***	676.30 ± 42.10**	487.00 ± 15.40
**Spermatocyte**	367.60 ± 43.70***	800.00 ± 44.20*	675.30 ± 32.40
**Spermatid**	521.33 ± 42.20**	1127.60 ± 137.20^Ns^	906.67 ± 89.40
**Spermatozoa**	225.50 ± 67.50***	913.30 ± 49.70 Ns	846.60 ± 35.50

Ns Not significant (*p > *0.05),

* Significant difference by *t*-test (*p* < 0.05),

** Highly significant (*p <* 0.01),

*** Extremely significant (*p < *0.001).

## Discussion

Techniques used to castrate horses include open, half-closed or modified open and closed.^2^^,^^17^ Most scrotal incisions are allowed to heal by second intention, although primary closure has been advocated.^9^^,^^18^ In our study, three techniques open (the scrotal wound was left unsutured), closed (the scrotal wound was sutured) and total ablation (complete excision of the scrotum) were used for unilateral orchidectomy, the time to healing and the postoperative complications were similar in the last two groups (sutured technique and total ablation technique). Each of the three techniques showed its advantages and disadvantages as mentioned in results, therefore the selection among the three techniques could be referred to the preference of the surgeon and the field where the castration will be performed because performing of un-sutured castration in the field or in hot areas is followed usually with complications of contamination of the scrotal wound with flies and debris and this technique will be more suitable for animals kept in clinic for a period of time. Suturing the scrotum after castration resulted in rapid healing process of the scrotal wound even with the swelling occurred after castration. This swelling was usually avoided with careful suturing of all underlying tissues in the scrotal wound, and subsided in few days following castration by massage of the swollen area, applying of a drain in the wound at the end of the suture line and with walking of the animal regularly in the days following castration. Total ablation of the testis and its surrounding tissues on its side was better when the surgeon intended to close the scrotal wound. The removal of the remaining skin and the underlying tissues in the scrotal wound decreased the size of the wound cavity to a large extent and the swelling occurred after castration was usually less than that with using the sutured technique. 

Complications that can arise following surgical castration include scrotal swelling or edema, excessive hemorrhage; septic funiculitis formation, septic peritonitis, omental herniation, incisional infections, hydrocele, penile trauma and evisceration (eventration).^19^^-^^21^ Except for peritonitis and evisceration, complications of castration are not considered life-threatening. In the present study, complications following UO varied according to the technique used but all complications were easily treated and controlled during the first week following castration except for a scrotal swelling that occurred in group II and III and needed longer time to subside. The removal of left testis resulted in compensatory effect on the right one which was removed after twelve weeks where its volume increased significantly. This might be due to increase in serum LH and FSH concentrations and perhaps, higher intratesticular testosterone.^4^ The reason could be explained because the UO increased the mean diameter of seminiferous tubules by 21% (*p* < 0.01) and of their lumina by 51%.^22^

The compensatory effect was also reflected on testis where the weight of the remaining testis increased significantly, this was similar to the result obtained by Barnes *et al*.,^23^ In the present study, epididymal weight was significantly increased by UO. This result was also recorded by Barnes *et al*.,^23^ The decrease of sperm motility and increase in abnormality percentages may be due to inflammatory reaction after removal of first testes.^24^^,^^25^

Histological examination of the testes of donkeys whose scrotum was sutured after unilateral orchidectomy (i.e. groups II and III) revealed hyperplasia of spermatogenic series and Leydig cells. Similarly, Putra and Blackshaw reported that UO was correlated with an increase in the number of Sertoli cells and germ cells and germ occupying the seminiferous epithelium. The compensatory hypertrophy primarily involved proliferation of Leydig cells and Sertoli cells.^26^

Testis and epididymal weight were increased linearly with age and after UO. Tubule diameter and epithelial height were greater in UO than in intact stallions. Testes of stallions underwent compensatory hypertrophy after UO.^27^ Unilateral orchidectomy increased the mean diameter of seminiferous tubules by 21% and of their lumina by 51% but did not significantly increase mean height of seminiferous epithelium or estimated length of seminiferous tubules per testis.^28^ The increase in the testicular weight of unilaterally castrated pigs was correlated with an increase in the number of Sertoli and germ cells at three months of age and germ cells at four months of age occupying the seminiferous epithelium. This was correlated with increased total seminiferous tubule length and larger cross-sectional area of the tubule. Sertoli cell occupancy did not differ significantly between unilaterally castrated and intact boars.^27^

In conclusion, unilateral orchidectomy could be performed successfully using different surgical techniques either closed or open. The surgical approach and technique used for UO depends on the clinical indications for surgery and the nature of the case. Primary closure technique is recommended to minimize local inflammation and swelling and thereby minimizing any effect they may have on the remaining testis. In addition, field castration is better performed using the suture technique or total ablation while for animals are kept in the clinic the three techniques could not show significant difference in advantages and disadvantages. Although UO results in compensatory effects on weight and volume of the remaining testis, its adverse effects on sperm values can affect the fertility of the animal.
